# KRAS as a Prognostic and Predictive Marker in Metastatic Non-Small Cell Lung Carcinoma: A Systematic Review

**DOI:** 10.7759/cureus.60061

**Published:** 2024-05-10

**Authors:** Sheereen Fatima, Nirav Pansuriya, Alisha Lakhani, Sai Madhuri, Reshma Ajmal, Ruchira Clementina, Zahabiya Lakdawala, Kinjal Shah, Husna Dilshana, Maya Andrea, Bejoi Mathew, Aashna Raheja

**Affiliations:** 1 Cancer Center, Kokilaben Dhirubhai Ambani Hospital and Medical Research Institute, Mumbai, IND; 2 Medicine, Surat Municipal Institute of Medical Education and Research, Surat, IND; 3 Research, Research MD, Vadodara, IND; 4 Medicine, Shantabaa Medical College And General Hospital, Amreli, IND; 5 Medicine, Indian Institute of Public Health, Hyderabad, IND; 6 Medicine, K.S. (Kawdoor Sadananda) Hegde Medical Academy, Mangalore, IND; 7 Medicine, Government Medical College, Nizamabad, Telangana, IND; 8 Medicine, C.U. (Chimanlal Ujamshibhai) Shah Medical College and Hospital, Surendranagar, IND; 9 Medicine, Robert Wood Johnson University Hospital, Rahway, USA; 10 Pathology, Al Azhar Medical College, Kumaramangalam, IND; 11 Medicine, American University of Integrative Science, Tucker, USA; 12 Internal Medicine, Sri Devaraj Urs Medical College, Kolar, IND; 13 Medicine, BGS Global Institute of Medical Sciences, Bengaluru, IND

**Keywords:** lung cancer, metastatic non-small cell lung carcinoma, kras mutation, predictive markers, prognostic markers

## Abstract

Metastatic non-small cell lung cancer (NSCLC) poses a significant clinical challenge, prompting a focused investigation into the role of KRAS mutations in prognosis and treatment response. Targeted therapies offer promising avenues for intervention, motivating a comprehensive analysis of existing evidence. Conducted in June 2023, our review delved into MEDLINE (Medical Literature Analysis and Retrieval System Online), Embase, Scopus, and the Cochrane Register of Controlled Trials. Rigorous inclusion and exclusion criteria guided the selection of 12 articles, comprising two randomized controlled trials (RCTs) and 10 observational studies. Multiple investigators independently executed data extraction, evaluating prognostic factors (overall and progression-free survival) and predictive outcomes (treatment and objective response). The Newcastle-Ottawa Scale (NOS) and modified Jadad scores were used for study quality assessment of observational studies and RCTs, respectively.

From an initial pool of 120 articles, the 12 selected studies, spanning 2013 to 2022, encompassed 2,845 metastatic NSCLC patients. KRAS mutations, particularly the G12C variant, emerged as a pivotal factor influencing treatment response. Notably, KRAS wild type patients displayed enhanced responses to platinum-based chemotherapy, while those with KRAS mutations exhibited favourable outcomes with immune checkpoint inhibitors (ICIs). The role of KRAS mutations as prognostic indicators in metastatic NSCLC is underscored by this systematic review, with implications for both survival and treatment response. The discernment between KRAS wild type and mutant patients offers insights into tailored therapeutic strategies, with platinum-based chemotherapy and immune checkpoint inhibitors emerging as context-dependent options. Nevertheless, more research is required to solidify the predictive role of KRAS and explore the efficacy of KRAS inhibitors and other targeted therapies, paving the way for refined and personalized interventions in the management of metastatic NSCLC.

## Introduction and background

Non-small cell lung cancer (NSCLC) is the most prevalent form of lung cancer, accounting for approximately 81-85% of all cases [1]. Metastatic NSCLC represents a devastating disease with significant treatment challenges and patient outcomes [2]. NSCLC encompasses various subtypes including adenocarcinoma, squamous cell carcinoma, and large cell carcinoma. KRAS mutations are more frequently observed in current or former smokers than in non-smokers. These mutations commonly occur as single-point mutations on exons 2 and 3 [3].

KRAS proteins, belonging to the RAS protein family, are small GTPases derived from the KRAS gene located on chromosome 12's short arm (12p)[4]. Through alternative splicing, this gene can produce two distinct proteins weighing around 21 KDa each: KRAS 4A and KRAS 4B. The most common KRAS mutation occurs when a single DNA base is substituted, resulting in amino acid substitution at positions 12, 13, or 61 of the KRAS protein [5]. Once activated, the KRAS protein disrupts the normal cell cycle and proliferation. This persistent activation of KRAS leads to the activation of downstream signalling pathways, such as MAPK and PI3K, which are crucial for regulating cell division, survival, and differentiation. Abnormal activation of these pathways due to KRAS mutations promotes uncontrolled cell growth, tumour initiation, progression, and metastasis in NSCLC.

However, recent advances in targeted therapies, particularly in KRAS G12C mutation, have shown promising results [6]. This review examines how KRAS mutations affect patient outcomes, treatment response, and survival in NSCLC. Understanding this correlation can guide precision medicine and inspire new therapeutic strategies.

## Review

Methodology

This review adheres to standard works in study design and statistical methods, primarily guided by Consolidated Standards of Reporting Trials (CONSORT) and Selective Amplification of Microsatellite Polymorphic Loci (SAMPL) guidelines.A systematic literature search of PubMed/MEDLINE (Medical Literature Analysis and Retrieval System Online), Embase, Scopus and Cochrane Register of Controlled Trials was conducted on June 24, 2023. We also looked through recent reviews manually to find more useful references.

Search Strategy

To develop an effective search strategy for our systematic review, Medical Subject Headings (MeSH) terms and keywords were combined using Boolean terms such as “AND”, “OR”, and “NOT”. The search used are given below.

Stage IV NSCLC: "stage IV NSCLC" OR “metastatic non-small cell lung cancer" OR ”advanced non-small cell lung cancer"

KRAS mutation: "KRAS mutation" OR "KRAS gene"

Predictive markers: "predictive marker" OR "biomarker"

Prognostic markers: "prognostic factor" OR "prognostic indicator" OR "prognostic outcome"

Study Selection

Inclusion criteria: Articles focusing on human subjects diagnosed with stage IV non-small cell lung cancer (NSCLC), specifically evaluating the presence or absence of KRAS mutation status and research exploring the predictive or prognostic value of *KRAS* mutation in terms of treatment response, survival outcomes, or disease progression were included. Only articles published in peer-reviewed journals, available in English, and comprising randomized controlled trials (RCTs) or observational studies are considered. Additionally, relevance to the research topic and accessibility of the full text were key inclusion factors.

Exclusion criteria: The exclusion criteria were chosen to ensure relevance, human clinical data, language, accessibility and rigorous research quality for focused analysis. Articles involving animals or in-vitro studies were excluded, as were those solely concentrating on early-stage NSCLC or other cancer types. Articles that did not assess the predictive or prognostic value of KRAS mutation status in stage IV NSCLC, articles not published in the English language, and reviews, letters to editors, audits, and conference presentations were excluded. Articles irrelevant to the research topic and those with non-availability of the full text are also excluded from consideration.

Two investigators independently found studies relevant to the topic and filtered out abstracts. If the abstract was considered suitable, we moved on to read the entire article. Another two investigators read the entire studies and reviewed and filtered them according to inclusion and exclusion criteria (Table 1). The two reviewers looked at all the articles independently to decide if they should be included. If they disagreed, they talked it out until they reached an agreement or they asked another reviewer to help. If there were multiple publications about the same trial, the one with the latest information was chosen.

**Table 1:** Inclusion and Exclusion Criteria NSCLC: non-small cell lung cancer

Serial Number	Inclusion Criteria	Exclusion Criteria
1.	Articles based on human subjects diagnosed with stage IV non-small cell lung cancer.	Articles on animals or in-vitro
2.	Articles evaluating the presence or absence of KRAS mutation status in patients with stage IV NSCLC	Articles that focus solely on early-stage NSCLC or other cancer types.
3.	Articles reporting on the predictive or prognostic value of KRAS mutation status in terms of treatment response, survival outcomes, or disease progression.	Articles not assessing the predictive or prognostic value of KRAS mutation status in stage IV NSCLC.
4.	Articles published in peer-reviewed journals and available in the English language	Articles not in English language
5.	Randomised controlled trials and observational studies	Reviews, letters to editor, audits and conference presentations
6.	Articles relevant to research topic and availability of full text	Articles irrelevant to research topic and non-availability of full text

Quality Assessment

Study quality was evaluated using the NOS for observational studies and modified Jadad scores for RCTs.

Data Collection

Two independent researchers gathered specific information from each study we included. For factors related to prognosis or prediction, all the results from both single-factor and multiple-factor analyses that were relevant to the outcomes we were interested in were collected. Prognostic factors, including overall survival (OS) and progression-free survival (PFS), were evaluated, while predictive outcomes were examined through treatment response and objective response assessments. Multivariable models were extracted to analyze the variation of covariates. The following were extracted when available: odds ratios (ORs), relative risks (RR), hazard ratio (HR), confidence interval (CI), p-values, the number and the proportion of events.

Results

Study Selection and Characteristics

**Figure 1:**
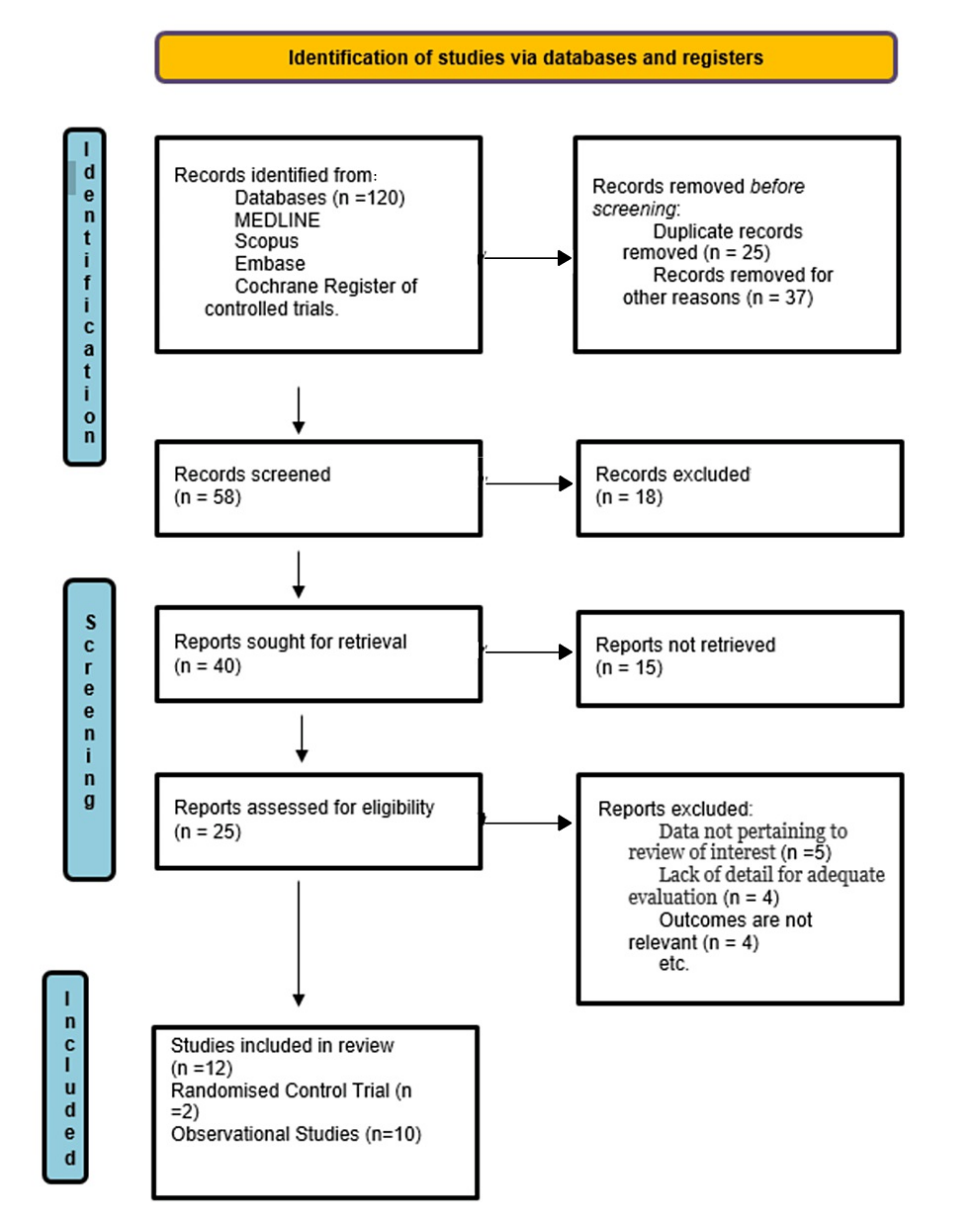
PRISMA Flowchart PRISMA: Preferred Reporting Items for Systematic Reviews and Meta-Analyses

Literature searches and title and abstract screening identified 120 articles for full-text examination, which subsequently led to the inclusion of 12 publications that were further categorized into RCTs (n=2) and observational studies (including retrospective and prospective cohort) (n=10). Most of the studies used were recent on the basis of their year of publishing, latest being in 2022 and the earliest being in 2013. The studies which qualified to be included in the final review were of both multivariate and univariate type, whereas three were only of multivariate type and two were solely of univariate type. Search screening and article shortlisting were performed using the Preferred Reporting Items for Systematic Reviews and Meta-Analyses (PRISMA) guidelines [7]. The PRISMA flowchart is given in Figure 1.

The chosen articles were based on studies conducted on human subjects with stage IV NSCLC, assessing the presence or absence of KRAS mutation, and reporting on the predictive or prognostic value of KRAS mutation in terms of OS, PFS, and treatment response [8-19].

Among the two RCTs, KRAS was only used as a prognostic marker. Of the 10 observational studies, four studies solely investigated whether KRAS could serve as a predictive factor, five studies looked at whether KRAS could tell us about the prognosis, and one study examined if KRAS could both predict and tell us about the prognosis.

Several studies have examined in detail the impact of KRAS mutations on clinical outcomes in patients with NSCLC. Li et al. conducted a Chinese single-centre retrospective study to examine the prognostic significance of KRAS mutations on immunotherapy outcomes in stage IV NSCLC patients [15]. Patients with KRAS-mutated tumours (KRAS-MT) showed significant improvements in both OS and PFS compared to those with KRAS wild-type tumours (KRAS-WT), as highlighted by the study. The study uncovered significant improvements in both OS and PFS when compared to patients in different conditions. Furthermore, the research underscored the profound influence of PD-L1 expression levels on survival outcomes, highlighting correlations that underscore the intricate interplay between KRAS status and immune dynamics.

Eklund et al. carried out a multicenter retrospective study in Sweden, focusing on the influence of both KRAS mutations and PD-L1 expression on standard first-line cancer treatments [10]. Their findings highlighted the adverse association between KRAS-MT presence and OS. Importantly, the study illuminated a distinct treatment response pattern wherein KRAS-MT patients exhibited more favourable outcomes with immune checkpoint inhibitor (ICI) treatment compared to platinum-based chemotherapy. This duality underscores the necessity of tailoring treatment strategies based on specific molecular characteristics.

Studies conducted across European countries also contributed significant insights into the role of KRAS mutations in NSCLC treatment outcomes. Noordhof et al. undertook a retrospective non-interventional study in the Netherlands, evaluating the prognostic value of KRAS mutations concerning ICI monotherapy [19]. Their comprehensive analysis unveiled a shorter median OS in patients with KRAS-MT compared to KRAS-WT counterparts. Importantly, this study provided a comprehensive overview of survival rates at different time points, aiding in understanding the disease trajectory.

Shifting the focus to targeted therapy, Janne et al. conducted a phase II single-arm clinical trial in the United States, investigating the impact of Adagrasib (MRTX849) on patients with KRAS G12C-mutated unresectable or metastatic NSCLC [20]. The study unveiled promising results, as evidenced by a notable objective response rate and favourable median OS. Intriguingly, the study's findings indicated that the predictive role of PD-L1 expression remained consistent across different patient subgroups, further emphasizing the potential of combination therapies for tailored treatments.

In China, Lei et al. embarked on a retrospective multi-city study to unravel potential associations between chemotherapy outcomes and specific KRAS-MT subtypes in stage III/IV NSCLC patients [8]. Their meticulous analysis demonstrated that patients harbouring the KRAS G12C mutation exhibited extended PFS following first-line chemotherapy. Moreover, intriguing trends emerged as pemetrexed-based chemotherapy yielded superior outcomes compared to taxane and gemcitabine-based regimens, further guiding treatment decisions.

Further exploration extended to assessing the impact of KRAS mutations within specific treatment contexts. Svaton et al. executed a single-centre retrospective study in Czechia, aiming to predict outcomes in patients receiving pemetrexed or docetaxel as second- or third-line therapy [9]. The study's results highlighted that KRAS-WT patients demonstrated superior median PFS and OS compared to patients with KRAS-MT, underscoring the complex interplay between mutation status and treatment response.

An Italian ancillary study within the TAILOR clinical trial, led by Marabese et al., focused on advanced epidermal growth factor receptor (EGFR)-WT NSCLC patients treated with platinum-based chemotherapy [12]. Their findings suggested that patients with KRAS-WT tumours experienced a higher median OS and a trend toward improved median PFS compared to those with KRAS-MTs, emphasizing the potential role of KRAS status in refining treatment strategies.

Notably, Brady et al. conducted a single-centre retrospective study in the United States to scrutinize the association between KRAS mutations and survival post-platinum doublet chemotherapy in stage IV lung adenocarcinoma patients [16]. In contrast to some previous findings, this study did not observe significant differences in OS or PFS between patients with KRAS-WT and KRAS-MT. This highlights the intricate nature of KRAS mutations' impact on treatment response, potentially influenced by diverse genetic backgrounds and treatment modalities.

Furthermore, the geographic context played a pivotal role in understanding KRAS mutation effects. A Danish single-centre prospective clinical trial by Nygaard et al. aimed to evaluate the prognostic value of the KRAS mutation across different stages of NSCLC [14]. Their comprehensive analysis revealed that patients with KRAS-MTs experienced lower chemotherapy response rates and shorter PFS compared to those with KRAS-WT tumours, adding depth to the understanding of KRAS's prognostic implications.

Lastly, Dingemans et al. conducted a multi-centre phase II single-arm clinical trial in the Netherlands, assessing the efficacy of sorafenib in KRAS-mutated patients pre-treated with platinum-based therapy, who had stage IIIb/IV NSCLC [17]. Their study demonstrated modest response rates, accompanied by median PFS and OS values. Interestingly, the study did not identify a significant correlation between specific KRAS mutant sub-mutations and treatment response, emphasizing the intricate and multifaceted nature of KRAS mutations in influencing treatment outcomes.

Table 1 gives a summary fo the included studies.

**Table 2:** Study design, stated objectives, study characteristics, arms/treatments, and salient outcomes of the included studies NSCLC: non-small cell lung cancer; EGFR: epidermal growth factor receptor: KRAS: Kirsten rat sarcoma oncogene; MT: mutant type; WT: wild type; OS: overall survival; PFS: progression-free survival; TKI: tyrosine kinase inhibitor; Pt: platinum; GI: gastrointestinal; AUC: area under the curve; ICI: immune checkpoint inhibitors; PD-L1: programmed death-ligand 1; AST: aspartate aminotransferase; ECOG: Eastern Cooperative Oncology Group; HR: hazard ratio; CI; confidence interval

Study	Study Design	Stated Objective/s	Genetic Characteristics	Arms/Treatments	Salient Outcomes	
Lei et al., 2019 [8]	Website-based self-reported retrospective multi-city study	To determine any association between chemotherapy outcomes and KRAS-MT subtypes in patients with Stage III/IV NSCLC	KRAS-MT: 100% Sub Mutations: G12C (33%) G12V (19%) G12A (12%) G12D (12%); Other identified KRAS mutations included G12S G12R G13C G13D G13S; Co-mutations TP53-KRAS (31%) EGFR-KRAS (11%) STK11-KRAS (8%)	1st line chemo: 1) Taxanes (9.3%), 2) Pemetrexed (68%), 3) Gemcitabine (4.3%), 4) Other (1.3%) 5)No (17.4%); 2nd line chemo: 1) Yes (42.7%), 2) No (57.3%)	In the univariate analysis, patients with the KRAS G12C mutation had longer duration of PFS after first-line chemotherapy than those with other KRAS mutations (4.7 vs. 2.5 months, p < 0.05); Pemetrexed-based chemotherapy tended to have better outcomes when compared to taxanes and gemcitabine-based regimens (PFS: 5.0 vs. 1.5 and 2.3 months, respectively, p > 0.07); When adjusted for age and smoking status, the KRAS G12C mutation and chemotherapy regimens remained positive impactors on PFS of the first-line chemotherapy only (p<0.01)	
Svaton et al., 2016 [9]	Single-centre retrospective	Investigate the role of KRAS mutation and specific KRAS mutations in prediction of outcome of patients with advanced NSCLC receiving pemetrexed or docetaxel as second- or third-line therapy	KRAS-WT (69.8%) KRAS-MT (30.2%) Sub Mutation A11P (2.6 %) G12A (7.7%) G12C (38.5 %) G12D (15.4%) G12R (0.0%) G12S (2.6%) G12V (7.7%) G13C (5.1%) G13D (5.1%) Unknown (17.9%)	Docetaxel or pemetrexed as second or third line treatments	KRAS-WT patients had a higher median PFS (2.3 months vs 1.6 months) and OS (16.1 vs 7.2 months)	
Eklund et al., 2022 [10]	Multicentre retrospective	To assess the impact of KRAS and PDL-1 expression on patients undergoing standard first-line cancer treatment for metastatic NSCLC	KRAS WT: 64.5% KRAS MT: 35.5% Sub Mutations: G12A: 6.8% G12B:40.3% G12D:11.2% G12V:20.9% Q61H:04.9% Others:16%	ICI Treatment: Pembrolizumab; Pt double treatment: Carboplatin/cisplatin and pemetrexed/ vinorelbine/ gemcitabine/paclitaxel/ etoposide/ vincristine	KRAS-MT presence was negatively associated with OS (p=0.014), a relationship which held true after Cox regression analysis; In KRAS MT patients undergoing first-line Pt treatment, the OS was significantly worse as compared to KRAS-WT cohort (median OS 9m vs 11m); KRAS-MT patients treated with ICI had a significantly (p = 0.003) better outcome compared to patients treated with Pt, with median OS of 23 vs. 9 months; KRAS-WT patients treated with ICI had significantly (p = 0.023) worse survival than patients treated with PT with a median OS of 6 vs. 11 months; KRAS-MT PD-L1(high) group had a significantly (p = 0.006) better outcome on ICB treatment compared to the KRAS-WT group, with a median OS of 23 vs. 6 months; KRAS-WT PD-L1(high) patients displayed a worse outcome among patients on ICB treatment compared to those on PT treatment (p = 0.010), with median OS of 6 months vs. 28 months for each respective treatment; At last follow-up, more of the KRAS-WT cohort alive (18.4% vs 13.6%)	
						Sun et al., 2013 [11]	Single-centre retrospective	To determine whether there is any significant difference in the treatment outcomes of various types of chemotherapy regimens stratified by KRAS mutation status in patients with stage IIIB/IV NSCLC and also to investigate the prognostic role of this biomarker.	KRAS-MT: 8% Sub Mutations G12D (33.3%) G12V (25.6%) G12C (23.0%) G12A (7.70%) G12S (2.60%) G12K (2.60%) G13D (2.60%) Q61H (2.60%) EGFR-MT (38%) Sub Mutations Exon 19 deletion (56.6%) L858R (33.5%) Exon 20M+ (6.0%) Exon 18M+ (3.3%) L747P in Exon 19 (0.56%) KRAS-WT and EGFR-WT (55%)	Pemetrexed-based regimen- Monotherapy-Plus Pt -First line -Second line -Third line; Gemcitabine Based regimen -Monotherapy -Plus Pt -First line -Second line -Third line; Taxane-based regimen -Monotherapy -Plus Pt -First line -Second line -Third line; EGFR TKI -First line -Second line -Third line	In the univariate analysis, women, relatively young patients (<65 years), non-smokers, patients with an adenocarcinoma diagnosis, relapsed disease after curative operation, KRAS-WT tumours, and EGFR-MT tumours were associated with longer OS. In the multivariate analysis, KRAS mutations (HR= 2.6, 95% CI: 1.8–3.7) and stage IV (HR = 1.8, 95% CI: 1.1–3.1) were independent predictors of poor prognosis, and EGFR mutations was an independent good prognostic factor (HR = 0.4, 95% CI: 0.3–0.5). When an OS curve is made comparing three cohorts (KRAS-MT + EGFR-WT, KRAS-WT + EGFR-MT, and KRAS-WT+ EGFR-WT) the curves differ significantly (p<0.01), with median OS for EGFR mutations > WT > KRAS mutations, a relationship which held true for a adenocarcinoma subgroup too. Efficacy of pemetrexed, gemcitabine, and TKI regimens was greater in the KRAS-WT group significantly, with higher PFS and response rates, while taxane based regimen had no significant effect on either cohort. The outcomes after EGFR TKI were vastly different according to EGFR mutation status: response rates (83% vs. 16%, p<0.001) and PFS (11.8 vs. 1.8 months, p<0.001) in the EGFR-MT vs. EGFR-WT groups	
Marabese et al., 2015 [12]	Ancillary study within the TAILOR clinical trial	To investigate the role of KRAS mutations in advanced EGFR-WT NSCLC patients treated with first-line Pt-based chemotherapy, and its effects on OS and PFS	KRAS WT (75.7%) KRAS MT (24.3%) Sub Mutations G12A (10%) G12C (43.3%) G12D (10%) G12F (3.3%) G12R (1.7%) G12S (1.7%) G12V (23.3%) G13C (3.3%) G13D (3.3%)	First second and third line chemotherapies, with any one of the two Pt agents (carboplatin/ cisplatin) and either Gemcitabine, vinorelbine, or pemetrexed for first-line, Docetaxel or erlotinib for second-line, Gemcitabine, vinorelbine, pemetrexed, docetaxel or erlotinib for third-line	Median OS was higher in KRAS-WT cohort vs KRAS-MT cohort (14.4 vs 10.6 months), with no difference when sub mutations are analysed. The median PFS, although statistically insignificant, was higher in the KRAS-WT cohort (7.2 vs 6.6)	
						Nygaard et al., 2013 [14]	Single-centre prospective clinical trial	To investigate the prognostic value of the KRAS mutation in patients with Stage II/III/IV NSCLC	KRAS-MT: 17.5% KRAS-WT: 82.5%	Carboplatin (dose based on AUC5, through IV at day 1 every three weeks) + vinorelbine (30 mg/m^2^ given through IV. at day 1 every three weeks and 60 mg/m2 p.o. day 8 every three weeks) for three weeks to a maximum of six cycles; Curative radiotherapy post-chemo, otherwise palliative radiotherapy	Significantly lower chemotherapy response rate in KRAS-MT cohort (p< 0.001); Significantly shorter PFS in KRAS-MT cohort (p=0.0043); Higher rate of disease control in KRAS-WT cohort (p<0.0001)	
Li et al., 2022 [15]	Single-centre retrospective	To investigate whether KRAS mutations could predict the effects of immunotherapy in patients with NSCLC Stage IV undergoing chemotherapy and immunotherapy	KRAS-WT (71.3%) KRAS-MT (28.7%) Sub Mutations: G12C (21.74%) G12V (21.74%) G12A (08.70%) G12D (17.39%) G12S (08.70%) G12C (08.70%) G12R (04.35%) Q61R (04.35%) Q61 (04.35%) -PD-L1 < 1% (32.5%) -PD-L1 ≥1% (67.5%)	4–6 cycles of pembrolizumab (2 mg/kg), carboplatin (AUC 5 or 6), and paclitaxel (200 mg/m^2^) for squamous or pemetrexed (500 mg/m^2^) for non-squamous every 3 weeks in first-line treatment.	The OS and PFS of the KRAS-MT were significantly greater than those of the KRAS-WT (p<0.05). No significant differences were observed in terms of 1- and 2-year survival rates between the KRAS-MT and KRAS-WT groups (87.0% vs. 66.7%, χ2=3.384, P=0.066; 56.5% vs. 38.6%, χ2=2.140, p=0.144). OS in the PD-L1-negative group was shorter than that in the PD-L1-positive group (15.2 vs. 20.6 months, p=0.009), while there were no significant differences in PFS between PD-L1-negative and PD-L1-positive groups among advanced NSCLC patients (5.6 vs. 10.7 months, p=0.214)	
Dingemans et al., 2013 [17]	Multi-centre Phase II Single-arm Clinical Trial	To investigate the efficacy of sorafenib in KRAS-MT, Pt-pretreated patients undergoing treatment for stage IIIb/IV NSCLC	KRAS-MT (100%) Sub mutations: G12A (5.3%) G12C (54.4%) G12D (14.0%) G12F (1.8%) G12S (1.8%) G12V (14.0%) G13A (1.8%) G13C (1.8%) G13Y (1.8%) Q61L (3.5%)	-Sorafenib 400 mg twice daily as oral tablets. If toxicities were reported, dosage refined from Level 0 (standard dose) to Level 1 (400 mg once daily) or Level 2 (400mg every other day). Treatment stopped if further refinement required or adverse events unresolved in 28 days post-interruption. Otherwise, on resolution, dosage allowed to step-up back to Level 0	-22.8% of patients stopped treatment before the first tumour response assessment because of reasons ranging from clinical deterioration, allergic reactions, and protocol violations. After 6 weeks under treatment, 8.8% had partial response, 43.8% had stable disease, and 47.4% registered progressive disease, with a 32-week median duration of response. There was no significant relation between any of the baseline characteristics and response. No significant relationship found between KRAS-MT submutations and sorafenib response. Median PFS and OS were 2.3 and 5.3 months respectively, with KRAS submutations having no bearing on both statistics. Sorafenib had adverse effects, mostly dermatological like hand-foot reactions and rashes but also GI and constitutional. No treatment related mortality	
Liyu and Gao, 2023 [18]	Single-centre retrospective	Analysing the prognostic effect of KRAS/P53 mutations on patients undergoing immune checkpoint inhibitor (ICI) therapy as second or later-line treatment for stage III/ IVB NSCLC. Analysing the predictive value of PD-L1 expression on outcomes in the same patient population when comparing the wild type and mutant type cohorts’	KRAS-WT or TP53-WT (72 %) KRAS-MT or TP53-MT (28%)	KRAS/ TP-53 MT cohort; ICIs alone (22.5%); ICIs and chemotherapy (60%) ICIs and antiangiogenesis agents (AAAs) (2.5%); ICIs, AAAs, and chemotherapy (15%) KRAS/TP53 WT cohort; ICIs alone (16.5%); ICIs and chemotherapy (61.2%); ICIs and antiangiogenesis agents (AAAs) (9.7%); ICIs, AAAs, and chemotherapy (12.6%)	Patients with KRAS/TP53-MT genes show a better response to immunotherapy and improved prognosis, with significant improvements in disease control rate (p=0.002), objective response rate (p<0.001), partial response rate (p<0.001), median PFS (p=0.003), and median OS (p=0.002). KRAS/TP53-MT and PD-L1 expressing patients had better prognosis when treated with ICIs, with patients with PD-L1 expression ≥1% showing significantly higher objective response rate(p < 0.001) and disease control rate (p = 0.004) rates compared with those in the KRAS/TP53-WT group	
Noordhof et al., 2021 [19]	Retrospective, non-interventional study with data from the Netherlands Cancer Registry	To assess the prognostic value of KRAS in relation to ICI monotherapy on a national scale in stage IV adenocarcinoma patients	KRAS-MT (56.8%), KRAS-WT (43.2%)	First-line pembrolizumab (100%)	OS at 90 days, 1 year and 2 years was 79% (95%CI 75–82), 57% (95%CI 53–61) and 44% (95% CI 39–48), respectively. The median OS was 17.2 months, and 19.2 months for patients with KRAS-MT vs16.8 months for KRAS-WT	

Assessment of KRAS Status

The total number of included patients was 2845. The RCTs had 304 patients and observational studies had 2541. Age was similar between trials, ranging from 58 to 71 years. There was a noticeable variation in race/ethnicity between trials. The male-to-female ratio varied significantly across different datasets. The highest male-to-female ratio was observed in two retrospective studies, with 71 males and nine females (ratio: 7.8) and 58 males and 17 females (ratio: 3.4) [8,9]. The overall male-to-female ratio across all datasets was approximately 1.21, indicating slightly more males than females in the combined data [8-12].

Seven of the 12 studies noted a significant correlation between smoking status and KRAS mutations, particularly with higher KRAS mutation rates observed in current smokers (OR: 2.7, 95%CI: 1.1-6.7) [8-14].

The most common techniques to detect KRAS mutations were polymerase chain reaction (PCR) based assays and direct sequencing of the genome of DNA. The other methods which were used to detect mutations in KRAS were paraffin section DNA extraction, quantitative PCR (qPCR) and next-generation sequencing (NGS), standard Giemsa staining, formalin-fixed paraffin-embedded, molecular genetic testing, denaturing capillary electrophoresis technique, KRAS by Sanger sequencing, and high-resolution melting analysis. Scorpion/ARMS technique was used for low-material samples [13].

The specific mutations studies were G12A, G12C, G12D, G12F, G12R, G12S, G12V, G13C, G13D, WT, mutated, A11P.The mutation found to be most common was G12C mutation [4-11].

KRAS as a Predictive Marker

The treatment received was studied and it was found that the participants in the 12 included studies received therapy ranging from platinum-based chemotherapy, platinum-based doublet chemotherapy (carboplatin or cisplatin along with pemetrexed or paclitaxel or docetaxel), sorafenib, and gemcitabine-based therapy to bevacizumab and ICIs (pembrolizumab) and adagrasib [12,13].

The studies showed that patients with KRAS-WT demonstrated a better response to platinum-based chemotherapy but had a poor response to ICBs. Conversely, patients with KRAS-MTs showed a better response to ICIs but had inferior outcomes after gemcitabine-based chemotherapy. KRAS-WT patients derived more benefit from bevacizumab treatment compared to those with KRAS-MT [14-20].

KRAS as a Prognostic Marker

Analysis of three retrospective studies and one RCT reported OS for patients with KRAS-WT versus mutant KRAS NSCLC. OS was found to be higher in KRAS-WT patients (28.9 months, 95%CI 14-28.9, p = 0.01) when treated with platinum-based chemotherapy + bevacizumab, whereas KRAS-MT patients exhibited OS of 23 months (p = 0.0061), 21 months (95%CI: 19.4-22.6), 19.2 months (p = 0.861, HR:1.03) in three retrospective studies when treated with ICI. Overall, OS did not have a significant effect on the KRAS-MT type in general.

Among the 12 studies, PFS was most prominent in retrospective studies involving mutated KRAS NSCLC treated with EGFR tyrosine kinase inhibitors (TKI) [11,15]. Among KRAS-WT patients, the use of bevacizumab in addition to platinum doublet therapy resulted in significant improvements in PFS (median 9.5 months vs. 4.8 months, p = 0.0041) [16]

Risk of Bias Assessment

Among the studies under examination, Liyu et al.'s emerges as a study of fair quality, revealing certain shortcomings within the domains of selection and outcome [18]. Notably, concerns arise regarding participant selection procedures, potentially influencing the study's internal validity. Furthermore, the assessment-of-outcome domain suggests potential issues with data measurement, implying a degree of susceptibility to bias. A notable omission is the lack of detailed blinding techniques, a factor of paramount importance in minimizing potential sources of bias.

Similarly, Li et al.'s study aligns itself with the fair quality category, demonstrating consistent fair ratings across all domains [15]. This conveys that while the study does not exhibit overt methodological flaws, there exists room for improvement in crucial facets such as participant selection, comparability, and outcome assessment. These weaknesses underscore the necessity for careful consideration when interpreting the study's findings, with an awareness of potential biases embedded within.

In resonance with Li et al. [15], Eklund et al.'s study occupies the fair quality classification, signifying analogous challenges across domains [10]. Although the study does not succumb to egregious biases, there exists an exigency for enhancement in pivotal aspects of the research process. This includes participant selection, comparability control, and outcome assessment.

The studies by Noordhof et al. [19], Svaton et al. [9], Brady et al. [16], and Sun et al. [11], ascend to the echelon of good quality, engendering confidence in their methodological rigour. These studies illustrate adept participant selection, comparability maintenance, and robust outcome assessment, collectively augmenting the dependability of their findings. It is evident that these studies are imbued with a degree of methodological integrity that enhances the veracity of their results.

Conversely, Lei et al.'s study presents a nuanced profile, excelling in the comparability domain but flagging in terms of representativeness and outcome assessment [8]. This implies potential limitations in the selection of participants and measurement of data. Researchers, therefore, are well-advised to consider the implications of these methodological nuances when evaluating the reliability of the study's conclusions.

Table 3 shows the risk of bias assessment by the NOS and Table 4 shows the tabulation of the modified Jadad score.

**Table 3:** Newcastle-Ottawa scale for risk of bias assessment Good quality: 3 or 4 stars in selection domain AND 1 or 2 stars in comparability domain AND 2 or 3 stars in outcome/exposure domain; Fair quality: 2 stars in selection domain AND 1 or 2 stars in comparability domain AND 2 or 3 stars in outcome/exposure domain; Poor quality: 0 or 1 star in selection domain OR 0 stars in comparability domain OR 0 or 1 star in outcome/exposure domain

Study	Selection				Comparability	Outcomes			Total
	Representativeness of exposed cohort	Selection of non-exposed cohort	Ascertainment of exposure	Outcome of interest absent at start of study		Assessment	Follow-up length	Adequacy of follow-up length	
Liyu et al. [18]	*	*	-	*	-	*	*	*	6
Li et al. [15]	*	*	*	*	*	*	*	*	8
Eklund et al. [10]	*	*	*	*	*	*	*	*	8
Noordhof et al. [19]	*	*	*	*	*	*	*	*	8
Lei et al. [8]	*	*	-	*	*	-	*	*	6
Svaton et al. [9]	*	*	*	*	*	*	*	*	8
Brady et al. [16]	*	*	*	*	*	*	*	*	8
Sun et al. [11]	*	*	*	*	*	*	*	*	8

**Table 4:** Modified Jadad score tabulated Scoring: Yes: +1; No:  0 In questions with a third ‘not described’ option, the ‘not described’ option is assigned a score of 0, while ‘No’ gets a score of -1

Items	Score			
	Jaane	Nygaard	Dingemans	Marabese
Was the study described as randomised in any way?	0	0	+1	+1
Was the method of randomisation appropriate?	0	0	+1	+1
Was the study described as being blinded/blinding?¹	0	0	0	0
Was the method of blinding appropriate?	0	0	0	0
Was there a description of withdrawals and dropouts?	+1	+1	+1	+1
Was there a clear description of the inclusion and exclusion criteria?	+1	+1	+1	+1
Was the method used to assess adverse effects described?	+1	+1	+1	+1
Was the method of statistical analysis described?	+1	+1	+1	+1
Total Score	4/8	4/8	6/8	6/8

Discussion

NSCLC is one of the most common causes of death related to cancers. Various studies have been conducted to assess the prognostic and predictive value of KRAS mutation in NSCLC; however, the result still remains unclear. Furthermore, it is believed that patients with KRAS mutants are resistant, or are poor responders, to EGFR-TKI. Therefore, there is a need to study deeper into this topic, as a result of which we have conducted this systematic review.

For evaluating the prognostic role of the KRAS gene in NSCLC, we have included 12 studies in our final assessment, two of which are RCTs, and the rest are observational studies. Unfortunately, the inability to access a few full-length articles limited us from reaching a strong standpoint. The results of this study must be analysed with utmost care as there is a possibility of increased bias in observational studies compared to RCTs while keeping in mind the heterogeneity of each patient that we might encounter in the clinical practice on a daily basis. The two RCTs reviewed were both multicentric studies, of which one assessed the outcome based on KRAS-MT and KRAS-WT while the other one assessed in terms of the patients who received sorafenib and those who did not [12,17].

The most common method used to detect the KRAS gene mutation in our study was PCR, followed by NGS, which we believe is a more suitable method for research and discovery purposes as it provides a broader view of the genomic landscape and is valuable in studying complex genetic changes [11-16]. The sample sizes in the studies included varied from as small as 57 to as large as 595, which is still a considerably small number to imply the results of this study to the general population. A study by Park et al. was excluded from our review as only one-third of the total participants (n= 1131) were tested [13]. 

Several studies reported different mutation frequencies for KRAS in advanced NSCLC. The highest frequency in our study was observed in Brady et al.'s study where the percentage of KRAS mutation stood at 41% [16]. These variations may be attributed to differences in patient populations, sample sizes, or geographical factors. Furthermore, when comparing mutation profiles across studies, we observed similar mutation types but varying frequencies. For instance, the prevalence of G12C mutations was consistently high in all studies, whereas the occurrence of G12V mutations showed variability. The mutations were commonly seen in codons 12, 13, 61 [16,17]. This observed variation in the KRAS mutations and their locations throws light on individualizing treatment strategies. Tailoring therapies based on specific mutation types and locations may improve treatment outcomes and patient prognosis in advanced NSCLC.

OS means the time from when treatment began until a person passed away, no matter what the cause of death was. If someone was still alive when we checked survival rates, we didn't count them. PFS is the time from when a person was randomly assigned to a treatment until the first sign of the disease getting worse or until they passed away, if that happened before the disease got worse. The OS and PFS outcomes in the included studies showed notable heterogeneity which may be attributed to differences in sample size, treatment regimen and the difference in the follow-up time. The best OS was observed in the study by Eklund et al. where KRAS mutated patients who were treated with immune ICI had a better outcome with OS being 23 months compared to KRAS mutated at nine months (p= 0.003) [10]. This was also observed in another study by Liu and Gao, which showed that a combined treatment regimen of ICI and first-line chemotherapy was significantly associated with prolonged OS (HR= 0.414; 95%CI 0.281-0.612; p<0.001) [18]. One study, by Noordaaf et al., observed that KRAS mutation had no prognostic influence with regard to pembrolizumab but the result was not significant (p= 0.86) [19].

Best PFS was observed in a study by Sun et al., which stated that KRAS treated with Erlotinib had a PFS of 17.7 months [11]. In the same way, a study by Li et al where KRAS treated with ICI and platinum-based chemotherapy had a PFS of 12.8 months [15]. On the other hand, a study conducted by Dingemans et al. stated that KRAS treated with platinum-based chemotherapy has a PFS of 2.3 months [17], while Sun et al. stated that KRAS treated with a pemetrexed-based regimen and a gemcitabine-based regimen has PFS of 2.1 months and 2.4 months, respectively [11].

Overall response rate (ORR) refers to the best outcome observed from the beginning of treatment until the disease either gets worse or comes back. ORR was poorly defined in the included studies. Dingemans et al. stated the ORR was 10.5% [17], while the study conducted by Sun et al. stated that ORR is good in KRAS-WT compared to KRAS-MT [11].

OS and PFS are statistically significant with KRAS-MT in all our studies except in two studies [11,19]. Follow-up therapeutic duration was not described in four studies whereas it was described in eight studies; it ranged from a minimum of 1 month to a maximum of 52.5 months. It should also be noted that only one of the observational studies compared KRAS mutant patients against patients who were double wildtype (KRAS, EGFR) and EGFR may act as a confounder; the remainder of the observational studies compared against a KRAS-WT [11] Only a few studies defined HR of OS and PFS, which concluded as a poor prognostic factor. Several studies described KRAS as either a prognostic or predictive factor except one retrospective cohort study, which described KRAS as both predictive as well as prognostic factors [11]. Various treatment modalities in different studies have been summarised in Table 2.

Ongoing research aims to develop targeted therapies called KRAS inhibitors to counteract the effects of KRAS mutations in cancer. These inhibitors show promise in cancer treatment, particularly for patients with KRAS-MT who may not respond well to traditional therapies. However, developing effective KRAS inhibitors is complex and challenging, requiring further investigation to better understand their potential and effectiveness in treating cancer patients with KRAS mutations [20].

Our study adds to the already existing evidence that KRAS by itself has either a negative role or no role in the prognosis of advanced NSCLC. In this study, KRAS gene mutation seemed to have a good predictive role in response to certain treatments received by the patients.

Limitations

There are some limitations with the included studies, which could make it harder to understand and draw conclusions from our review. First, different studies used different methods to test for KRAS, and sometimes they didn't explain them very well. Also, the way they reported their results varied; some mentioned HRs without giving a p-value, while others did include it. Plus, some studies that could have looked at how KRAS affects outcomes didn't give the p-value for that interaction. Another issue was that we didn't have enough studies with control groups; however, we have to consider that it's difficult and not ethical to run placebo-controlled trials with cancer patients.

## Conclusions

This systematic review suggests that the presence of KRAS mutations could be linked to worse outcomes in terms of survival and response to treatment among patients with metastatic NSCLC. Additionally, it emphasizes the importance of further research to solidify KRAS's role as a predictive factor. There's a clear need for more published studies to delve deeper into the use and effectiveness of KRAS inhibitors and other targeted therapies. By conducting more comprehensive studies, we can gain a better understanding of how KRAS influences the course of the disease and how targeted treatments can potentially improve outcomes for patients with NSCLC.
